# Construction and characterization of *Bs*GDH-CatIB variants and application as robust and highly active redox cofactor regeneration module for biocatalysis

**DOI:** 10.1186/s12934-022-01816-2

**Published:** 2022-06-02

**Authors:** Kira Küsters, Ronja Saborowski, Christian Wagner, Rebecca Hamel, Jan-Dirk Spöring, Wolfgang Wiechert, Marco Oldiges

**Affiliations:** 1 Institute of Bio- and Geosciences IBG-1: Biotechnology, Forschungszentrum Jülich GmbH, 52425 Jülich, Germany; 2 Institute of Biotechnology, RWTH Aachen University, 52074 Aachen, Germany; 3 Aachen Biology and Biotechnology (ABBt), RWTH Aachen University, 52074 Aachen, Germany; 4 Computational Systems Biotechnology (AVT.CSB), RWTH Aachen University, 52074 Aachen, Germany

**Keywords:** Catalytically active inclusion bodies, Immobilization, Protein aggregates, Protein engineering, Downstream processing, Enzymes

## Abstract

**Background:**

Catalytically active inclusion bodies (CatIBs) are known for their easy and cost efficient production, recyclability as well as high stability and provide an alternative purely biological technology for enzyme immobilization. Due to their ability to self-aggregate in a carrier-free, biodegradable form, no further laborious immobilization steps or additional reagents are needed. These advantages put CatIBs in a beneficial position in comparison to traditional immobilization techniques. Recent studies outlined the impact of cooperative effects of the linker and aggregation inducing tag on the activity level of CatIBs, requiring to test many combinations to find the best performing CatIB variant.

**Results:**

Here, we present the formation of 14 glucose dehydrogenase CatIB variants of *Bacillus subtilis*, a well-known enzyme in biocatalysis due to its capability for substrate coupled regeneration of reduced cofactors with cheap substrate glucose. Nine variants revealed activity, with highest productivity levels for the more rigid PT-Linker combinations. The best performing CatIB, *Bs*GDH-PT-CBDCell, was characterized in more detail including long-term storage at −20 °C as well as NADH cofactor regeneration performance in repetitive batch experiments with CatIB recycling. After freezing, *Bs*GDH-PT-CBDCell CatIB only lost approx. 10% activity after 8 weeks of storage. Moreover, after 11 CatIB recycling cycles in repetitive batch operation 80% of the activity was still present.

**Conclusions:**

This work presents a method for the effective formation of a highly active and long-term stable *Bs*GDH-CatIB as an immobilized enzyme for robust and convenient NADH regeneration.

**Supplementary Information:**

The online version contains supplementary material available at 10.1186/s12934-022-01816-2.

## Background

Catalytically active inclusion bodies (CatIBs) can be used as alternative enzyme immobilization strategy with high application potential. In contrast to other enzyme formulations, CatIBs reveal many advantages like low cost production, easy purification and a biologically produced, carrier-free as well as biodegradable immobilization technology without the need for additional carrier material or reagents [1–3]. Inclusion bodies (IBs) were long seen as inactive and misfolded protein aggregates. However, for some enzymes the formation of natural CatIBs, like lysine decarboxylase of *Escherichia coli *(*Ec*LDC*c*), adenosine 5′-monophosphate phosphorylase of *Thermococcus kodakarensis* and lipase A of *Bacillus subtilis* were reported. Instead of inactive inclusion bodies, the protein aggregates retained some catalytical activity [4–6]. Enzymatic activity of such natural CatIBs or of enzymes which do not form any IBs can be enhanced or enforced by generating synthetic tailor-made CatIBs. Different kinds of linker and aggregation inducing tags can be fused to the enzyme of interest to improve the activity of the resulting CatIB. Recent studies revealed that different combinations of linkers and aggregation inducing tags lead to cooperative effects and activity level variations for each specific enzyme. For example, *Ec*LDCc showed higher activity levels in combination with the rigid PT-Linker instead of the flexible SG-Linker [4]. For alcohol dehydrogenase of *Ralstonia sp*. the combination of the 3HAMP-Tag led to higher k_cat_ values than the TDoT-Tag [7, 8]. Therefore, when testing new enzymes for CatIB formation, a set of different linker/aggregation inducing tag combinations need to be constructed and tested, since systematic structure–function relationship for CatIBs is still to be explored.

Glucose dehydrogenase of *Bacillus subtilis* (*Bs*GDH, EC 1.1.1.47) can be used to convert β-d-glucose to β-d-glucono-1,5-lactone. During this reaction *Bs*GDH reduces the oxidized cofactor NAD^+^ to its reduced form NADH [9], which is of high value as reducing reagent for biocatalytic transformations. Therefore, *Bs*GDH can be used as an in-situ cofactor regeneration system, which is essential for industrial application, in order to perform low-cost cofactor recycling of NADH. The use of glucose (0.3 € kg^−1^) as a substrate is much more cost-efficient than using NADH (184 k € kg^−1^) in equimolar amounts. So far, NADH regeneration was performed with the soluble form of GDH, co-immobilized in a nanofiltration membrane reactor or in form of self-assembling all-enzyme hydrogels [10–12]. The use of CatIBs for NADH recycling provides an efficient alternative solution showing cost effective production and further advantages, such as long-term storage options, high enzyme stability and simple reusability in repetitive process applications [1], making them the perfect candidates to investigate a novel approach for NADH regeneration in repetitive process application.

Here we report the construction and characterization of a set of *Bs*GDH-CatIB variants. The plasmids encoding the CatIBs were generated via Golden Gate Assembly with a set of two linkers and eight aggregation inducing tags. While two combinations failed in the molecular biology construction, fourteen variants were successfully constructed and characterized regarding morphology and specific volumetric productivity. The best performing CatIB, *Bs*GDH-PT-CBDCell, was characterized in more detail including long-term storage at −20 °C as well as NADH cofactor regeneration performance in repetitive batch experiments with CatIB recycling.

## Results and discussion

### Production of CatIBs and microscopic analysis

**Fig. 1:**
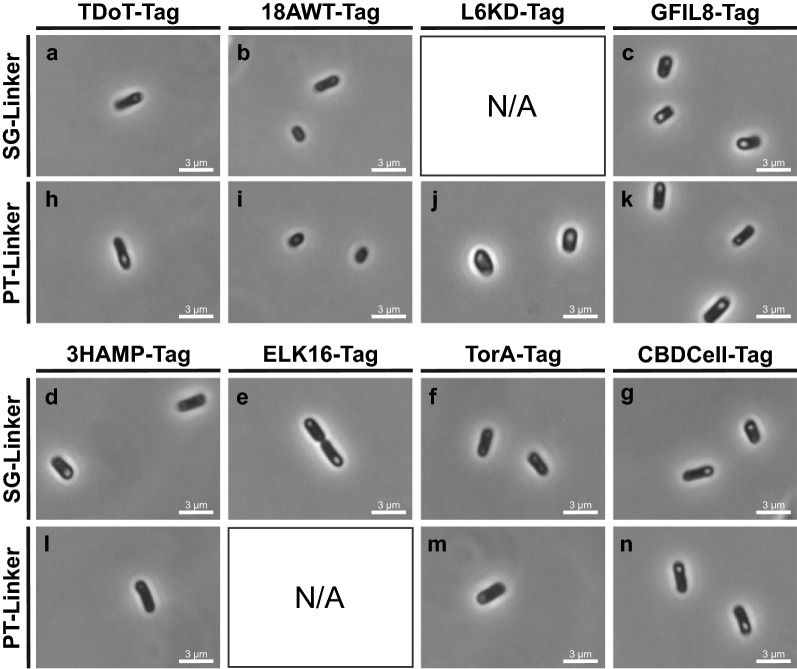
Microscopic analysis of *E. coli* BL21(DE3) cells producing *Bs*GDH-CatIB variants at 1000-fold magnification. The microscopic images were taken after cultivation for 3 h at 37 °C followed by 69 h at 15 °C in M9-AI*.* **a** *Bs*GDH-SG-TDoT, **b** *Bs*GDH-SG-18AWT, **c** *Bs*GDH-SG-GFIL8, **d** *Bs*GDH-SG-3HAMP, **e** *Bs*GDH-SG-ELK16, **f** *Bs*GDH-SG-TorA, **g** *Bs*GDH-SG-CBDCell, **h** *Bs*GDH-PT-TDoT, **i** *Bs*GDH-PT-18AWT, **j** *Bs*GDH-PT-L6KD, **k** *Bs*GDH-PT-GFIL8, **l** *Bs*GDH-PT-3HAMP, **m** *Bs*GDH-PT-TorA, **n** *Bs*GDH-PT-CBDCell

The glucose dehydrogenase of *Bacillus subtilis* (*Bs*GDH; EC 1.1.1.47), was C-terminally fused with either a flexible SG- or a more rigid PT-Linker as well as one out of eight aggregation inducing tags (TDoT, 18AWT, L6KD, GFIL8, 3HAMP, TorA, CBDCell and ELK16; see Additional file 1: Table S1) [1, 6, 13–19]. *Bs*GDH has a homotetrameric quaternary structure with free C- and N-termini. In this study, the 16 possible linker/aggregation inducing tag combinations were fused to the C-terminus of the *Bs*GDH and tested for enzymatic activity. Fourteen *Bs*GDH-CatIB variants were successfully generated and analyzed in more detail. The fourteen CatIB variants were produced in *E. coli* BL21(DE3) using M9 autoinduction medium (See Additional file 1: Table S2). Phase contrast microscopy with a 1000-fold magnification was used to verify the formation of *Bs*GDH-CatIBs in this host (see Methods). Like typical IBs, CatIBs can be found at the cell poles as granule-like structures or refractive particles (Fig. 1) [20]. All strains were screened via microscopic analysis to observe the CatIB formation and the influence on the cell and CatIB morphology.

The microscopic analysis of the *Bs*GDH-CatIB variants confirmed the hypothesis that combinations of different linkers and aggregation inducing tags lead to different cell and IB morphologies [4]. The variants with PT-L6KD and SG-ELK16 formed dense inclusion bodies with 63% and 75% of the cells carrying CatIBs, respectively (Fig. 1, Table 1). Whereas the cells with SG-ELK16 revealed an *E. coli* like cell structure, the cells carrying the CatIB with PT-L6KD appeared in an untypically round cell structure. Very similar IB and cell morphologies were found with both linkers and aggregation inducing tags, but the overall yield of cells carrying CatIBs showed large differences. While for CBDCell (SG: 75%; PT: 78%) and GFIL8 (SG: 63%; PT: 75%) more than 50% of the cells showed CatIBs, TorA (SG: 32%; PT: 23%), TDoT (SG: 17%; PT: 13%) and 3HAMP (SG: 16%; PT: 6%) resulted in values from 6 to 32% only. Whilst PT-18AWT did not form any IBs, the corresponding SG-18AWT variant displayed only 1% IB carrying cells. Interestingly, the same phenomenon was shown for the 18AWT-Tag with *Ec*LDCc and strengthened the hypothesis that this tag has a higher tendency to bind to cell membrane components rather than forming isolated IBs [4, 21]. Moreover, comparing microscopic analysis data of *Bs*GDH with previous results from *Ec*LDCc revealed similar CatIB and cell morphology effects, such as abnormal cell structures in combination with L6KD-Tag or a looser CatIB structure with 3HAMP-Tag, but it is less dominant with *Bs*GDH than with *Ec*LDCc. Strikingly, the influence of the linker/aggregation inducing tag combination on CatIB formation was different between the two enzymes. With *Ec*LDCc-SG-TDoT 78% of the cells carried CatIBs, whereas with *Bs*GDH-SG-TDoT it was only 17% [4]. This strongly supports the hypothesis that there is no optimal linker/aggregation inducing tag combination, but rather a more complex interaction between the three parts, i.e., including the nature of the target protein in focus.

**Table 1:** Protein production and productivity data of *Bs*GDH wild type and *Bs*GDH-CatIB variants

	CatIBs visibility (SDS) (Yes/No)	CatIBs visibility (Microscopy) (Yes/No)	Cells with CatIBs [%]	Pellet activity (Yes/No)	P_v_ Pellet (g L^-1^d^-1^)	Specific P_v_ Pellet (g L^-1^d^-1^ g_CatIB_^-1^)	P_v_ Supernatant (g L^-1^d^-1^)
*Bs*GDH_WT_	No	No	0	No	0	0	76.8
*Bs*GDH-PT-TorA	No	No	0	No	0	0	1.9
*Bs*GDH-PT-CBDCell	Yes	Yes	52	Yes	19.0	55.8	0.3
*Bs*GDH-PT-3HAMP	Yes	Yes	6	Yes	12.6	48.3	0.8
*Bs*GDH-PT-GFIL8	Yes	Yes	75	Yes	6.7	26.8	1.3
*Bs*GDH-PT-L6KD	Yes	Yes	63	Yes	19.6	36.3	5.4
*Bs*GDH-PT-18AWT	No	No	0	No	0	0	11.3
*Bs*GDH-PT-TDoT	Yes	Yes	13	Yes	16.3	24.3	11.3
*Bs*GDH-SG-ELK16	Yes	Yes	75	Yes	2.3	7.9	0
*Bs*GDH-SG-TorA	No	Yes	32	No	0	0	24.7
*Bs*GDH-SG-CBDCell	Yes	Yes	75	Yes	0.6	2.6	0
*Bs*GDH-SG-3HAMP	Yes	Yes	16	Yes	1.0	3.2	3.6
*Bs*GDH-SG-GFIL8	Yes	Yes	63	No	0	0	0
*Bs*GDH-SG-18AWT	Yes	Yes	79	No	0	0	1.3
*Bs*GDH-SG-TDoT	No	No	0	Yes	1.4	6.4	11.2

Pellet activity was tested by adding 200 mM glucose, 0.4 mM NAD^+^ and 40 mM TAE buffer (pH 7) to the CatIB pellet fraction after purification. Productivity was calculated after 20 min (pellet fraction), 12 min (supernatant; *Bs*GDH-CatIBs) or 6 min (supernatant *Bs*GDH_WT_) reaction time. Microscopic images were taken after main cultivation (72 h) and analyzed regarding the number of cells that carry CatIBs (n ≥ 45).

P_V_: volumetric productivity (g_NADH_ L^−1^ d^−1^ g_CatIB_^−1^)

### Downstream processing and specific volumetric productivity of fourteen BsGDH-CatIB variants

A high overall protein yield together with high enzymatic activity are important CatIB features. A previously improved purification protocol [4] was used for simple and fast CatIB purification. The purified CatIBs were divided in several identical 1 mL aliquots. Three of these aliquots were used for the CatIB weight determination and one or three others were used for activity measurement. Moreover, presence of CatIBs was verified via sodium dodecyl sulfate- polyacrylamide gel electrophoresis (SDS-PAGE, Additional file 1: Figure S1). The SDS gel revealed respective bands for all *Bs*GDH-CatIB variants in the insoluble pellet fraction, except for the variants with PT-18AWT, PT-TorA and SG-TorA (Table 1).

**Fig. 2:**
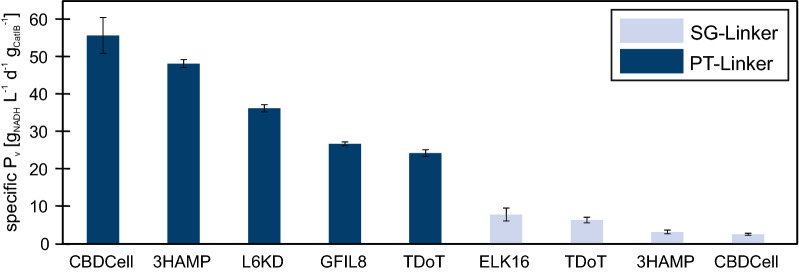
Comparison of specific volumetric productivity of fourteen *Bs*GDH-CatIBs. The enzyme assay was performed at 37 °C, 15 min, 1000 rpm, 1 mL in 40 mM TAE buffer (pH 7) with 200 mM glucose and 0.4 mM NAD^+^. CatIB weight was determined after drying the CatIBs at 80 °C for 24 h, followed by additional 24 h in a desiccator. n_t_ = 3 for PT-CBDCell and SG-ELK16; all others: n_a_ = 3

After CatIB purification and SDS-PAGE analysis, an enzymatic activity assay was performed in 40 mM TAE buffer (pH 7) using 200 mM glucose as a substrate and 0.4 mM NAD^+^ as the cofactor. The reproducibility of the CatIB purification and enzyme assay workflow was tested in technical triplicates with *Bs*GDH-PT-CBDCell and *Bs*GDH-SG-ELK16 (Fig. 2). The other CatIB variants have been tested in analytical triplicates. In general, a sufficiently good reproducibility of the technical and analytical replicates was observed with relative standard deviation of 8.6–21.8% and 2.2–15.4%, respectively. The errors of the technical replicates were found to be slightly higher, since they include the pure analytical error as well the pipetting and processing steps. The specific volumetric productivity (specific P_v_) of all variants was determined based on NADH conversion after 20 min reaction time. A comparison of the specific P_v_ showed that nine out of the fourteen CatIB variants revealed activity, with the 18AWT- and TorA-Tag with both linkers and the GFIL8-Tag with PT-linker showing no activity. Strikingly, the five highest specific P_v_ values were all reached with the more rigid PT-Linker combinations. This strengthens the hypothesis from Küsters et al. 2021 that the linker selection has an enormous impact on the CatIB performance as indicated by the PT-Linker leading to higher specific P_v_ values with the *Bs*GDH-CatIB data. The rigidity of the PT-Linker seems to have an important influence on the CatIB structure–function relationship. However, although the PT-Linker again led to higher productivity, the ranking of the best aggregation inducing tags differed between the two enzymes. Interestingly, only a few cells with *Bs*GDH-PT-3HAMP formed visible CatIBs (6%), but this variant is the second best CatIB variant in specific P_v_. This indicates that 3HAMP seems to favor more loose CatIB structures, which were not fully visible under the microscope. This effect was already shown in [19] where a combination with 3HAMP resulted in high activity levels for benzaldehyde lyase from *Pseudomonas fluorescens*, but without revealing visible CatIBs in light microscopy.

### Enzymatic activity vs morphology of *Bs*GDH-CatIB variants

The comparison of CatIB visibility on microscope pictures as well as on SDS gels with the productivity levels of the CatIBs revealed suitable and less suitable *Bs*GDH-CatIBs. Contrary to a few other enzymes, the control, *Bs*GDH wild type, did not form natural CatIBs [4–6]. Despite being highly expressed from the same plasmid construct as the CatIB variants, it seems that the wild type enzyme showed a typical soluble protein behavior which is given by: (I) no insoluble fragment on SDS gel, (II) no visible IBs on microscopic images, (III) no activity in the pellet fraction after purification and (IV) activity in the supernatant (76.8 g_NADH_ L^−1^ d^−1^). The CatIB variants showed clear differences between the linker/aggregation inducing tag combinations. The two aggregation inducing tags, TorA and 18AWT, only formed inactive IBs. Only SG-18AWT revealed a band in the insoluble fraction of the SDS gel, meaning that the other variants did not form insoluble protein or the level was too low to be verified via SDS-PAGE. In case of TorA, the formation of visible CatIBs was proven with microscopic images (PT: 23% and SG: 32%). However, it may happen that these insoluble proteins return to their soluble form during the purification procedure. This hypothesis might be strengthened by the fact that the supernatant after cell lysis of *Bs*GDH-SG-TorA has the highest productivity level (24.7 g_NADH_ L^−1^ d^−1^) of all CatIBs. Moreover, *Bs*GDH-SG-GFIL8 did not reveal any pellet activity, which made this variant together with 18AWT and TorA variants irrelevant for further analysis. In comparison to these inactive variants, nine of fourteen variants revealed enzymatic activity in the pellet fraction. The productivity level differed between the variants from 2.6 g_NADH_ L^−1^ d^−1^ g_CatIB_^−1^ (*Bs*GDH-SG-CBDCell) up to 55.8 g_NADH_ L^−1^ d^−1^ g_CatIB_^−1^ (*Bs*GDH-PT-CBDCell). A previous study showed that *Ec*LDCc in combination with the PT-L6KD showed the highest activity whereas the combination with SG-Linker showed the lowest activity [4]. The same effect was shown for *Bs*GDH-CatIBs, where SG-CBDCell revealed the lowest productivity level and *Bs*GDH-PT-CBDCell the highest. Moreover, *Bs*GDH-PT-CBDCell was also the variant that revealed the highest number of CatIB producing cells (78%). Nevertheless, the comparative analysis of microscopic image data and activity of all variants showed that a high number of CatIB forming cells alone is not predictive of high activity levels. For example *Bs*GDH in combination with PT-GFIL8, SG-ELK16 and SG-CBDCell all revealed 75% CatIB producing cells. However, the productivity levels of these CatIB variants differed between 26.8 g_NADH_ L^−1^ d^−1^ g_CatIB_^−1^ (*Bs*GDH-PT-GFIL8), 7.9 g_NADH_ L^−1^ d^−1^ g_CatIB_^−1^ (*Bs*GDH-SG-ELK16) and 2.6 g_NADH_ L^−1^ d^−1^ g_CatIB_^−1^ (*Bs*GDH-SG-CBDCell). Taking all together, the most important results were (I) variants without visible CatIBs on microscopic images and on SDS gel can be excluded from further analysis, (II) microscopic images alone cannot be used to confirm successful CatIB formation, (III) the linker is one decisive factor for high activity and (IV) *Bs*GDH-PT-CBDCell is the best performing CatIB for *Bs*GDH.

### CatIB storage condition test with *Bs*GDH-PT-CBDCell

**Fig. 3:**
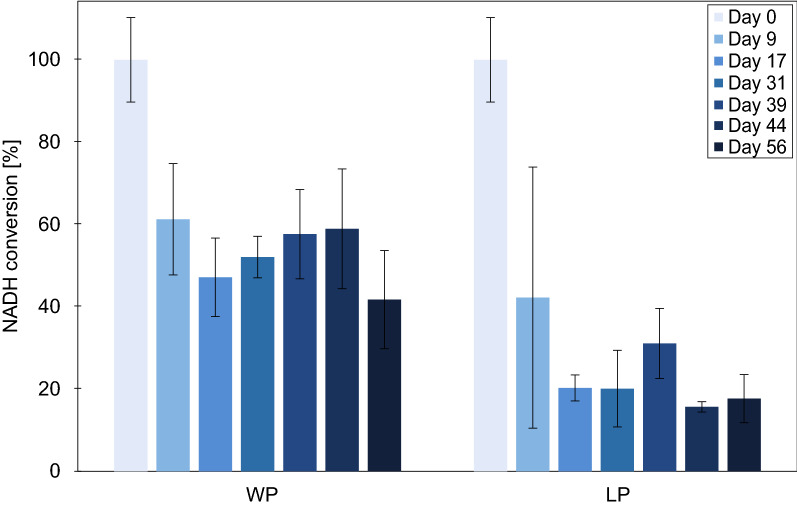
Storage stability testing of *Bs*GDH-PT-CBDCell. The wet pellets (WP) were directly frozen after purification or first lyophilized (LP) before storing them in the freezer at – 20 °C. Technical triplicates were tested. The enzyme assays were performed at 37 °C, 15 min, 1000 rpm, 1 mL in 40 mM TAE buffer (pH 7) with 200 mM glucose and 0.4 mM NAD^+^. A reference assay was performed at day 0, before freezing or freeze-drying. The other assays were performed at day 9, 17, 31, 39, 44 and 56

The best *Bs*GDH-CatIB, *Bs*GDH-PT-CBDCell, was used to characterize potentially suitable long-term storage conditions. In previous studies, lyophilization of CatIBs was used for long-term storage [1, 7, 13]. In this study, the long-term storage of lyophilized pellets (LP) and wet pellets (WP) was compared. Whereas WP were directly stored at −20 °C, LP were first lyophilized (Fig. 3). A reference assay was performed immediately after purification at the same day (day 0) to visualize the activity loss of the CatIB due to initial freezing and lyophilizing step. This reference value was set to 100%. After storing WP and LP at −20 °C for 9, 17, 31, 39, 44 and 56 days, the CatIBs were tested for their activity. Whereas the WP showed an initial loss of approx. 40%, LP revealed an initial loss in the range of about 60% in comparison to the non-frozen reference sample at day 0 directly after purification. Interestingly, in the LP and WP samples from day 9 to 56, there is no clear trend to lower activity values, indicating that the first step of lyophilisation or freezing is the most harmful step. It seemed that freezing the WP directly without lyophilization led to higher remaining activity of the CatIB and lyophilization did not show a positive effect during the investigated time frame. Apart from the loss of activity due to the treatment before −20 °C storage, both methods led to stable CatIB activity levels over the entire time span. In contrast to the *Bs*GDH-CatIB data, a hydroxynitrile lyase CatIB of *Arabidopsis thaliana* revealed higher activity levels when using it in lyophilized form [13]. This indicated that the best storage condition could also differ between various CatIB enzymes.

### CatIB and NADH recycling with BsGDH-PT-CBDCell

**Fig. 4:**
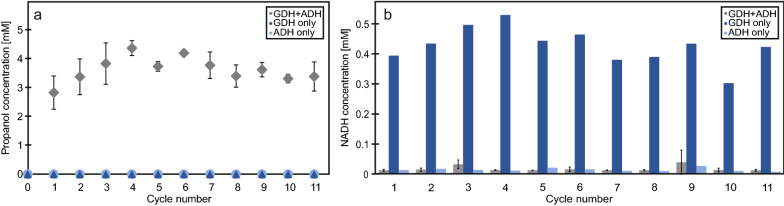
Repetitive batches for the production of propanol from propanal with soluble *Sc*ADH and *Bs*GDH-CatIBs for NADH recycling. **a** Measured propanal concentration with GC. **b** NADH concentration was measured with a photometer. *Bs*GDH-CatIBs, purified from 0.5 mL cells with OD_600nm_ 20, were reused for 11 cycles. 11.5 U *Sc*ADH were freshly added with 40 mM TAE (pH 7), 200 mM glucose, 20 mM propanal, 0.4 mM NAD in each cycle. The final reaction volume was 0.5 mL. Each reaction was performed at 37 °C for 30 min. GDH + ADH samples were measured in technical triplicates

Based on the promising results from previous experiments, the best CatIB candidate, *Bs*GDH-PT-CBDCell, was also used to test the stability and robustness during repetitive batch process application. This nicely showcased the (I) biocatalytic performance of the *Bs*GDH-CatIB to efficiently regenerate NADH from its oxidized form NAD^+^ and (II) the high stability of the CatIB variant during recycling in repetitive batch process. For this, propanal reduction with NADH to propanol catalyzed by the alcohol dehydrogenase from *Saccharomyces cerevisiae* (*Sc*ADH) was coupled with NADH cofactor regeneration enabled by *Bs*GDH-PT-CBDCell CatIB oxidation of glucose to gluconate. To set up the repetitive batch on a 0.5 mL scale, 200 mM glucose, 0.4 mM NAD^+^, 20 mM propanal, and 11.5 U *Sc*ADH was used. Each of the eleven repetitive batch cycles was performed with the recycled CatIBs for 30 min. For the glucose/propanal ratio a tenfold excess of glucose was chosen. In order to avoid any issue with propanal substrate volatility, it was used at 20 mM, which was approx. 4 times higher than the expected conversion to the product propanol. Reaction monitoring of the biocatalytic process was done by gas chromatography measurement of the propanol concentration (Fig. 4a) and spectrophotometric measurement of the NADH concentration (Fig. 4b).

The repetitive batch experiment revealed the cooperative activity of the *Bs*GDH-PT-CBDCell CatIB and the *Sc*ADH, as shown by the formation of propanol (Fig. 4a; GDH+ADH). The control experiments with *Bs*GDH-PT-CBDCell CatIB and *Sc*ADH alone did not show any propanol. This was expected since in the case of *Sc*ADH only NAD^+^ is provided and no NADH is present here. For the sole use of *Bs*GDH-PT-CBDCell CatIB no propanol formation was found, due to the lack of *Sc*ADH. Nevertheless, *Bs*GDH-PT-CBDCell CatIB activity could be verified in the controls proven by NADH concentration in the range of approx. 0.4 mM which was formed from the NAD^+^ present (Fig. 4b; GDH only). Interestingly, the data for propanol and NADH concentration were slightly increasing during the first 4 cycles (Fig. 4a, b). This effect may have occurred because of already bound NAD^+^ in the reused *Bs*GDH-CatIBs or some activation effect during the initial cycles. The experiments with *Bs*GDH-PT-CBDCell CatIB to regenerate NADH from NAD^+^ and the *Sc*ADH show the biocatalytic reaction from propanal to propanol with in-situ regeneration of reduced cofactor NADH. Interestingly, the NADH level stays low during the whole reaction time, indicating that the NADH regeneration was the rate limiting step in the reaction sequence and that overall reaction was not completed. This is supported by the estimation of overall propanal conversion to be approx. 15–20% based on 20 mM initial propanal substrate concentration and about 3–4 mM propanol product after each 30 min reaction cycle. As a consequence based on 0.4 mM initial NAD + concentration, each cofactor molecule was regenerated approx. 7.5–10 times. In order to optimize the biocatalytic reaction more *Bs*GDH-PT-CBDCell CatIB could be used in order to increase the regeneration rate of NADH and initial NAD + might be also reduced. Since there is simple access to *Bs*GDH-PT-CBDCell CatIB preparations with option for stable long term storage at −20 °C for at least 8 weeks, this seems to be a convenient and ready-to-use NADH cofactor regeneration module for biocatalytic conversions. The data of the eleven repetitive batch processes seem to indicate highly stable NADH regeneration enabled by the *Bs*GDH-PT-CBDCell CatIBs and one could speculate that many more reaction cycles would have been possible. Moreover, the aliquots of the lyophilized or directly frozen CatIB preparations could be added during the process, in case enzymatic activity needs to be increased or if a specific limiting NADH regeneration rate should be realized.

## Conclusion

Although microscopic and SDS-PAGE analysis alone cannot be used to make predictions about the CatIB productivity levels, these methods are important tools to do a pre-sorting of successful CatIB generation, especially when analyzing many CatIB variants in a short time. Strains without any visible IBs on the microscopic images in combination with missing bands in the insoluble fraction on the SDS gel can be directly excluded from further analysis to save resources and time.

Nine out of fourteen *Bs*GDH-CatIBs revealed glucose dehydrogenase activity. The most efficient *Bs*GDH CatIB was *Bs*GDH-PT-CBDCell showing effective CatIB formation in 78% of the *E. coli* cells in combination with the highest specific P_v_ value (55.8 g_NADH_ L^−1^ d^−1^ g_CatIB_^−1^). As mentioned in previous studies, the more rigid PT-Linker led to higher specific P_v_ values than the SG-Linker, which strengthened the hypothesis that the linker has a decisive influence on the CatIB activity [4]. Nevertheless, it was also shown that the aggregation inducing tag has a significant influence on the activity, which seems to strictly depend on the enzyme used. These results indicate, when generating CatIBs with new proteins of interest, many linker/aggregation inducing tag combination needs to be tested to find not only successful CatIB variants with some enzymatic activity, but linker/aggregation inducing tag combinations with high performance applicable for robust biocatalytic processes.

Additionally, *Bs*GDH-PT-CBDCell CatIBs showed a high long-term storage stability at -20 °C and also high stability in solution, as has been demonstrated in repetitive batch experiments for NADH recycling with commercial *Sc*ADH.

In comparison to traditional GDH technologies, like using soluble or immobilized enzyme, which can either be used only once or several times requiring additional immobilization or filtration steps, *Bs*GDH-CatIBs enable a simple self-immobilization technology in combination with convenient reusability with high rates. The achieved NADH regeneration rates in the range of 2 g_NADH_ L^−1^ h^−1^ g_CatIB_^−1^ provide attractive performance. With these features, especially *Bs*GDH-PT-CBDCell would be suitable as a NADH regeneration module in future biocatalytic research and industrial application.

## Methods

### Reagents and chemicals

All chemicals were purchased from ROTH (Karlsruhe, Germany) and Merck (Sigma-Aldrich, Missouri, USA), unless stated otherwise. Enzymes for molecular biology were purchased from New England Biolabs GmbH (Frankfurt am Main, Germany).

### Construction of expression plasmids

The synthetic gene of the *Bs*GDH, the SG- and PT-Linker as well as the eight aggregation inducing tags, TDoT-, 18AWT-, L6KD-, GFIL8-, 3HAMP-, TorA-, CBDCell-, ELK16-Tag, were synthesized by Synbio Technologies (Monmouth Junction, New Jersey, USA). The synthetic sequences contained *Bsa*I recognition and restriction sites needed for Golden Gate Assembly. The synthetic gene encoding for *Bs*GDH was assembled with one of the two linkers, one of the eight aggregation inducing tags as well as the so-called suicide plasmid in a ratio of 1:1:1:3 during Golden Gate Assembly. The suicide plasmid served as the expression plasmid backbone. It consisted of a pET28a vector with an integrated *ccdB* gene, coding for the CcdB toxin, which is lethal for standard *E. coli* strains, like *E. coli* DH5α and *E. coli* BL21(DE3). It functioned as an accurate Golden Gate Assembly control with zero-background cloning [22]. During Golden Gate Assembly, the *ccdB* gene was removed by *Bsa*I and the CatIB-Linker-Tag sequence was inserted by the T4-Ligase. After *E. coli* DH5α was transformed with the Golden Gate Assembly mixture, only strains carrying the successful CatIB plasmid were able to grow while strains carrying the original vector were killed due to the produced toxin. To start Golden Gate Assembly, 2.5% (v/v) T4-Ligase as well as 2.5% (v/v) *Bsa*I restriction enzyme, was added to the mixture. The Golden Gate Assembly was performed in a PCR cycler (37 °C, 5 min and 16 °C, 5 min—15 cycles; 65 °C, 20 min). Information about all plasmids that were used in this study are summarized in Additional file 1: Table S1, Supporting Information. The final expression plasmids were sequenced and verified for the correct assembly (Eurofins GmbH, Hamburg, Germany).

### Protein production, cell disruption and protein purification

CatIB production was performed by cultivating *E. coli* BL21(DE3) carrying the respective expression plasmids in M9 autoinduction medium (See Additional file 1: Table S2). The cultivation was performed after a modified protocol by Lamm et al. 2020, 1 L shaking flasks were used with a filling volume of 100 mL and a shaking frequency of 170 rpm (Infors HT Multitron Standard, Infors AG, Bottmingen, Switzerland). The main cultivation was inoculated with an OD_600nm_ of 0.05 of an overnight culture in LB complex medium with additional kanamycin (37 °C, 170 rpm). The standard incubation procedure included two phases. The first growth phase included 3 h at 37 °C, followed by 69 h at 15 °C to produce active CatIBs. The optical density of the main cultures was determined to perform a normalization of the cell cultures to OD_600nm_ = 10 unless otherwise stated. The purification process was continued with 30 mL cell suspension with the specific optical density. The cells were harvested by centrifugation at 5,000 x*g* for 10 min (Beckman GS15-R, Beckman Coulter, Krefeld, Germany). The next centrifugation step (3 min at 5000×*g*) was performed after washing the cell pellet with 20 mL of 0.9% NaCl solution. The supernatant was discarded. 2.7 mL cell lysis buffer, BugBuster^®^ HT Protein Extraction Reagent (Merck KGaA, Darmstadt, Germany) with the addition of 0.146 g L^−1^ lysozyme, was added to the cell pellet to perform cell lysis. The suspension was incubated at 20 °C for 20 min and 750 rpm. After cell lysis the soluble and insoluble protein fraction were separated by centrifugation at 5000 x*g* for 30 min. The pellet was washed with 20 mL Milli-Q^®^ water followed by centrifugation. 30 mL Milli-Q^®^ water was added to the CatIB pellet and 1 mL aliquots in 1.5 mL Eppendorf tubes were made. A centrifugation step was performed and the supernatant was discarded (10,000 x*g* for 5 min). The pellets were used for enzyme assay or for weight determination. The CatIB weight was determined after drying the pellet at 80 °C for 24 h, followed by a second 24 h step in a desiccator. The enzymatic assay samples were stored on ice at 4 °C overnight and was used for enzyme activity measurements on the next day.

### Sodium dodecyl sulfate-polyacrylamide gel electrophoresis (SDS-PAGE)

For SDS-PAGE analysis sample preparation was performed by adding 2×Laemmli sample buffer to a purified CatIB-water suspension and to the soluble fraction originated from a cell culture with a normalized OD_600nm_ of 10 (See Protein production, cell disruption and protein purification). After incubation for 10 min at 95 °C, the samples were applied to Criterion™ 4–12% Bis-Tris protein gel, 1.0 mm, with 18 wells (Bio-Rad Laboratories GmbH, Feldkirchen, Germany) together with a protein marker (PageRuler Prestained Protein *ladder*, ThermoFisher Scientific). Gel electrophoresis was performed in NuPAGE™ MES SDS running buffer (1x) at 200 V, 500 mA and 150 W. The gel was stained with Simply Blue™ SafeStain for 1 h.

### Microscopic analysis

Phase-contrast microscopic analysis was performed for *E. coli* BL21(DE3) strains with CatIB formation strains. 1 µL of each sample was given on a microscope slide and covered with a coverslip. The microscope slide was positioned upside down on the desk of an inverted Nikon Eclipse Ti2 microscope (Nikon GmbH, Düsseldorf, Germany). The sample was observed with a CFI Plan Apo Lambda 100X Oil objective (Nikon GmbH, Düsseldorf, Germany) and images were taken with a Thorlab camera DCC154M-GL (Thorlabs Inc., Newton, New Jersey, USA). The amount of cells that carried CatIBs was counted by analyzing at least 50 cells per CatIB variant.

### Activity assay

Enzyme activity testing was performed by adding 40 mM TAE buffer (pH 7), 200 mM glucose and 0.4 mM NAD^+^ to the CatIB pellet, originated from a cell culture with a normalized OD_600nm_ of 10 (See Protein production, cell disruption, and protein purification). The soluble fraction, after cell lysis, was refilled with TAE-glucose-NAD^+^ ratio compared to the CatIB pellet fraction. The samples were incubated at 1000 rpm and 37 °C. Samples were taken after 0, 3, 6, 12 and 20 min and the enzyme was inactivated by adding 80% (v/v) methanol. The inactivated samples were diluted 1:10 with 40 mM TAE buffer, before the NADH fluorescence was analyzed photometrically in an UV microtiter plate (pro infinite m200, Tecan, Männedorf, CH). NADH was measured with an excitation wavelength of 340 nm and an emission wavelength of 470 nm. A calibration curve of different NADH concentrations was measured with the same parameters.

### Storage stability experiment

Four 100 mL main cultivations of *E. coli* BL21(DE3) with *Bs*GDH-PT-CBDCell CatIBs were produced in 1 L shaking flasks. After cultivation, they were combined and the OD600nm was adjusted to a value of 10. The samples were purified with the same protocol as before (See protein production, cell disruption and protein purification). The first half of the purified CatIBs was divided in 1.5 mL reaction tubes with 1 mL CatIBs each with a following centrifugation step at 10,000 x*g* for 5 min. The supernatant was discarded, before the samples were stored at −20 °C as wet pellets (WP). The other half of the samples were first lyophilized at −54 °C and 0.01 mbar for 24 h before storing them at −20 °C as lyophilized pellets (LP). The reference samples (day 0) were tested via activity assay in triplicates (See activity assay) directly after purification. The storage stability of WP and LP were tested after 9, 17, 31, 39, 44 and 56 days at −20 °C (See activity assay).

### NADH/CatIB recycling experiment

50 mL main cultivation of *E. coli* BL21(DE3) with *Bs*GDH-PT-CBDCell CatIBs were produced in 1 L shaking flasks. After cultivation they were adjusted to OD600nm 20. The samples were purified with the same protocol as before (See protein production, cell disruption and protein purification). The purified CatIBs were divided in 1.5 mL reaction tubes with 1 mL CatIBs each. The activity of the CatIBs was tested in repetitive batches. While the CatIBs were reused for 11 cycles, 0.5 mL of the reaction solution, containing 40 mM TAE buffer (pH 7), 11.5 U *Sc*ADH (Sigma-Aldrich: A3263_150KU), 20 mM propanal, 200 mM glucose and 0.4 mM NAD^+^, were freshly added after each cycle. The reaction was performed for 30 min at 37 °C and 40 µL samples were inactivated with 160 µL methanol. The samples were prepared and NADH levels were measured as before (See activity assay). Moreover, the propanol concentration of the samples was analyzed via GC.

### GC analysis

Samples were analyzed using a gas chromatograph (Thermo TRACE_1300_1310, Thermo Scientific, Waltham, USA) with a CP-Chirasil-Dex CB (Agilent, Santa Clara, USA) column (i.d. 0.25 mm; 25 m × 0.25 µm), starting at 40 °C, hold for 0.5 min, followed by a gradient of 10 °C/min to 77 °C which was held for 0.75 min. Detection was done by FID with propanol showing a retention time of 4.13 min.

## Supplementary Information

**Additional file 1:** **Table S1: **Plasmids used in this study. **Table S2**: Recipe of M9 Autoinduction medium—1000 mL **Figure S1: **Evaluation of CatIB formation by SDS-PAGE analysis. After cultivation, the optical density of the cultures were normalized to OD600nm = 10. The cells were disrupted and the crude cell extract was separated by centrifugation into the soluble and the insoluble CatIB-containing pellet fraction. The pellet fraction was washed with Milli-Q water. The pellet samples were 1:1 diluted with SDS sample buffer and 15 μL of each samples was loaded onto the gel. The gel was stained with SimplyBlue™ SafeStain.

## Data Availability

All data generated or analyzed during this study are included in this article and its Additional file 1.

## Abbreviations

ADH: Alcohol dehydrogenase
CatIB: Catalytically active inclusion body
GDH: Glucose dehydrogenase
P_v_: Volumetric productivity
SDS-PAGE: Sodium dodecyl sulfate polyacrylamide gel electrophoresis
