# Supplementary material for: Construction and characterization of BsGDH-CatIB variants and application as robust and highly active redox cofactor regeneration module for biocatalysis
Source: Microb Cell Fact. 2022 Jun 2;21:108. doi: 10.1186/s12934-022-01816-2 (PMC9161568; doi:10.1186/s12934-022-01816-2)
Supplement: Supplementary file 1 — Additional file 1: Table S1: Plasmids used in this study. Table S2: Recipe of M9 Autoinduction medium—1000 mL Figure S1: Evaluation of CatIB formation by SDS-PAGE analysis. After cultivation, the optical density of the cultures were normalized to OD600nm = 10. The cells were disrupted and the crude cell extract was separated by centrifugation into the soluble and the insoluble CatIB-containing pellet fraction. The pellet fraction was washed with Milli-Q water. The pellet samples were 1:1 diluted with SDS sample buffer and 15 μL of each samples was loaded onto the gel. The gel was stained with SimplyBlue™ SafeStain. [file 12934_2022_1816_MOESM1_ESM.pdf]

Construction and characterization of *Bs*GDH-CatIB variants and application as robust and highly active redox cofactor regeneration module for biocatalysis

Kira Küsters[1,2], Saborowski Ronja[1], Wagner Christian[1], Hamel Rebecca[1], Spöring Jan-Dirk[1,2], Wolfgang Wiechert[1,4], Marco Oldiges[1,2*]

[1:]Institute of Bio- and Geosciences IBG-1: Biotechnology, Forschungszentrum Jülich GmbH, 52425 Jülich, Germany

[2:]Institute of Biotechnology, RWTH Aachen University, 52074 Aachen, Germany

[3:]Aachen Biology and Biotechnology (ABBt), RWTH Aachen University, 52074 Aachen, Germany

[4:]Computational Systems Biotechnology (AVT.CSB), RWTH Aachen University, 52074 Aachen, Germany

*: Corresponding author: m.oldiges@fz-juelich.de

ORCID-Identifier

Kira Küsters: https://orcid.org/0000-0002-2472-9926

Jan-Dirk Spöring: https://orcid.org/0000-0002-5656-3537

Wolfgang Wiechert: https://orcid.org/0000-0001-8501-0694

Marco Oldiges: https://orcid.org/0000-0003-0704-5597

Supporting Information

Table S1: Plasmids used in this study.

Vector	Genotype
pET28a	*ColE1 lacZ'* Kan^R P_{T7} P_{lac}
pET28a::CcdB	*ColE1 lacZ'* Kan^R P_{T7} P_{lac} *ccdB*
pET28a::*Bs*GDH::SG::TDoT	*Bs*GDH::SG::TDoT fragment in pET28a backbone
pET28a::*Bs*GDH::SG::18AWT	*Bs*GDH::SG::18AWT fragment in pET28a backbone
pET28a::*Bs*GDH::SG::GFIL8	*Bs*GDH::SG::GFIL8 fragment in pET28a backbone
pET28a::*Bs*GDH::SG::3HAMP	*Bs*GDH::SG::3HAMP fragment in pET28a backbone
pET28a::*Bs*GDH::SG::ELK16	*Bs*GDH::SG::ELK16 fragment in pET28a backbone
pET28a::*Bs*GDH::SG::TorA	*Bs*GDH::SG::TorA fragment in pET28a backbone
pET28a::*Bs*GDH::SG::CBDCell	*Bs*GDH::SG::CBDCell fragment in pET28a backbone
pET28a::*Bs*GDH::PT::TDoT	*Bs*GDH::PT::TDoT fragment in pET28a backbone
pET28a::*Bs*GDH::PT::18AWT	*Bs*GDH::PT::18AWT fragment in pET28a backbone
pET28a::*Bs*GDH::PT::L6KD	*Bs*GDH::PT::L6KD fragment in pET28a backbone
pET28a::*Bs*GDH::PT::GFIL8	*Bs*GDH::PT::GFIL8 fragment in pET28a backbone
pET28a::*Bs*GDH::PT::3HAMP	*Bs*GDH::PT::3HAMP fragment in pET28a backbone
pET28a::*Bs*GDH::PT::TorA	*Bs*GDH::PT::TorA fragment in pET28a backbone
pET28a::*Bs*GDH::PT::CBDCell	*Bs*GDH::PT::CBDCell fragment in pET28a backbone

Table S2: Recipe of M9 Autoinduction medium – 1000 mL

Salt Stock solution (5x)	200 mL
$MgSO_4*7H_2O$ solution (246.48 g L^{-1})	1 mL
$CaCl_2*5H_2O$ solution (14.702 g L^{-1})	1 mL
Trace element solution (1000x)	1 mL
Citrate/Fe solution<br>(7.5 g L^{-1} $FeSO_4*7H_2O$<br>113.95 g L^{-1} tri-NaCitrat*$2H_2O$)	2 mL
Thiamin solution (10 g L^{-1})	1 mL
2 % (w/v) Lactose solution	100 mL
5 % (w/v) Glucose solution	10 mL
Glycerin 99%	4 mL
Kanamycin solution (50 g L^{-1})	1 mL
add Milli-Q (final volume)	1000 mL
**Salt Stock (5x)**	1000 mL
$(NH_4)_2SO_4$	25 g

KH_2PO_4	15 g
Na_2HPO_4	33.9 g
NaCl	2.5 g
NH_4Cl	10 g
add Milli-Q (final volume)	1000 mL
**Trace elements (1000x)**	1000 mL
$AlCl_3*6H_2O$	0.75 g
$CoCl_2*6H_2O$	0.6 g
$CuSO_4*5H_2O$	2.5 g
H_3B_3	0.5 g
$MnSO_4*1H_2O$	17.1 g
$Na_2MoO_4*2H_2O$	3 g
$NiCl_2*6H_2O$	1.7 g
$ZnSO_4*7H_2O$	15 g
Dissolve in 100 mL Milli-Q and 50 mL 32% HCl and add Milli-Q to final volume	

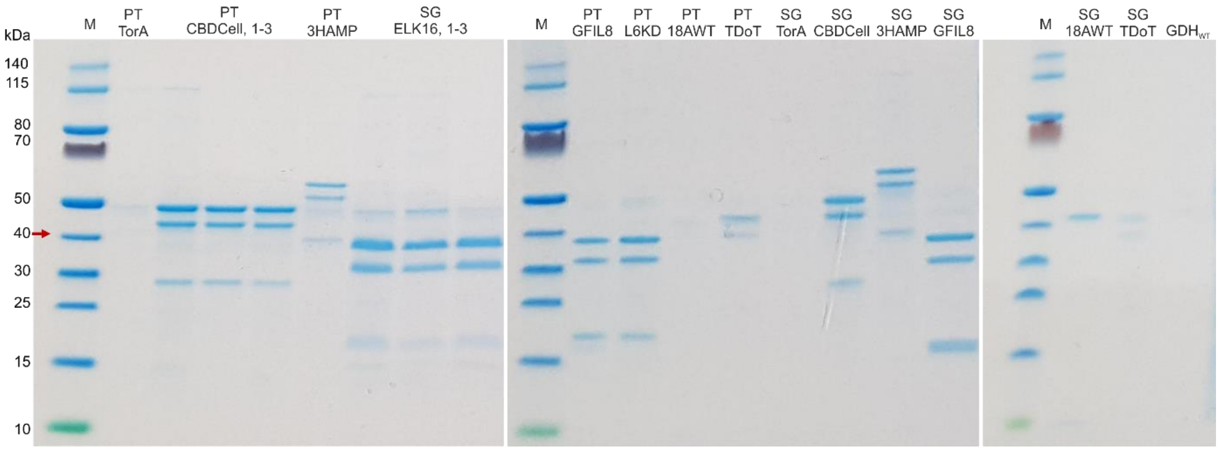

Figure S1: Evaluation of CatIB formation by SDS-PAGE analysis. After cultivation, the optical density of the cultures were normalized to OD_{600nm} = 10. The cells were disrupted and the crude cell extract was separated by centrifugation into the soluble and the insoluble CatIB-containing pellet fraction. The pellet fraction was washed with Milli-Q water. The pellet samples were 1:1 diluted with SDS sample buffer and 15 µL of each samples was loaded onto the gel. The gel was stained with SimplyBlue™ SafeStain.
